# Behaviour of twin- and triplet-born lambs and their dam 3 to 18 hours after birth is not a useful predictor of lamb survival to weaning

**DOI:** 10.5713/ajas.19.0479

**Published:** 2019-11-12

**Authors:** G. V. Gronqvist, R. E. Hickson, P. R. Kenyon, S. T. Morris, K. J. Stafford, R. A. Corner-Thomas

**Affiliations:** 1 School of Agriculture and the Environment, Massey University, Palmerston North 4410, New Zealand

**Keywords:** Twin, Triplet, Maternal Recognition, Survival, Behaviour, Lamb

## Abstract

**Objective:**

An experiment was designed to determine if behaviour traits expressed by twin- and triplet-bearing lambs and their dams at 3 to 18 hours of age (after the immediate ewe-lamb bonding had occurred) were associated with lamb survival to weaning.

**Methods:**

The behaviour of twin and triplet lambs and their dams was assessed in the paddock at 3 to 18 hours after birth. Observations were made of the number of high- and low-pitched bleats, time to stand, make contact with dam, suck from dam and follow dam were recorded for each lamb. The maternal behaviour score of each dam was assessed. A random sub-sample of lambs were assessed during a maternal-recognition test at 12 or 24 hours of age. Traits included time spent standing, sitting, walking, time taken to reach the ewes and time spent with the ewes as well as the number of high- and low-pitched bleats emitted by the lamb.

**Results:**

In the paddock, for each additional second required for twin-born lambs to follow their dam, lambs were 1.004 (95% confidence interval [CI] 1.000 to 1.008) times more likely to survive to weaning (p<0.05). The opposite relationship, however, was seen in triplet lambs. For each additional second required for triplet-born lambs to follow their dam, lambs were 0.996 (95% CI 0.993 to 0.999) times as likely to survive to weaning (p<0.05). During the maternal recognition test, twin-born lambs were 0.989 (95% CI 0.979 to 1.000) times as likely to survive to weaning for every additional second they took to reach the contact zone (p<0.05). Similarly, triplet-born lambs were 0.994 (95% CI 0.989 to 0.999) as likely to survive for every additional second they took to reach their dam (p<0.05).

**Conclusion:**

All ewe behaviours and the majority of lamb paddock and test behaviours were not associated with the survival of twin- or triplet-born lambs and, therefore, are of little use as indicators of lamb survival to weaning.

## INTRODUCTION

Lamb mortality is of concern to the New Zealand sheep industry from both an economic and an animal welfare perspective [1]. New Zealand studies conducted in outdoor pastoral conditions have reported lamb losses from birth to weaning of 14% to 20% for twin-born lambs and 28% to 56% for triplets [2]. An understanding of the factors involved in multiple-born lamb mortality could improve ewe flock productivity through the development of strategies to reduce lamb mortality. Ewe and the lamb behaviours during the first few days post-birth can affect lamb survival [3–5]. In order to survive, the neonatal lamb must exhibit key behaviours including: standing soon after birth, sucking soon after standing, following the dam closely and returning to her if separated [4–6]. The time required for the lamb to progress through this series of behaviours immediately after birth has been used to predict the likelihood of lamb survival [6–8]. Lamb survival is also affected by ewe behaviour post-parturition [9,10].

The vast majority of the studies examining the impacts of ewe and lamb behaviour on lamb survival have reported observations made immediately after birth [6–8,11]. Such observations are achievable in housed sheep, however, the nature of extensive pastoral farming conditions in many parts of the world make observations immediately post-birth impractical. Furthermore, such observations that occur during the establishment of the ewe-lamb bond can result in the disturbance of extensively grazed ewes thus interfering with the ewe-lamb bonding and the ewe rejecting her lamb(s) [12]. Observation of neonatal lamb behaviour in extensively managed sheep, therefore, should be conducted after the ewe-lamb bond has been established.

If a behaviour trait that is displayed in the hours post-birth could be identified as a predictor of lamb survival it could be used as a tool to identify lambs that require intervention to improve their chances of survival. In addition, it could be used as a research tool to evaluate the effects of various ewe interventions in pregnancy on lamb survival. Therefore, the aim of the current study was to determine if behaviour traits expressed by twin- and triplet-bearing ewes and their lambs at 3 to 18 hours of age (after the immediate ewe-lamb bonding had occurred) were associated with lamb survival.

## MATERIALS AND METHODS

These experiments were conducted with approval of the Massey University Animal Ethics Committee (MUAEC 09/55, 10/21, 10/41, 11/38, 12/21, and 12/50). All experiments were conducted during consecutive years on Massey University’s Keeble Farm, 5 km south of Palmerston North, New Zealand (40°S, 175°E).

### Animals and treatments

Four hundred and twenty-three twin-bearing ewes and their 846 lambs and 278 triplet-bearing ewes and their 834 lambs were included in this experiment. These ewes were a subset of those enrolled in a series of seven experiments examining the impact of body condition and nutrition during pregnancy on ewe and lamb performance and behaviour (Table 1). All studies utilised Romney ewes as they are the most common breed type in New Zealand [13]. The ewes included in the present analyses were those that had complete sets of twin- and triplet-born lambs that were alive when the behaviours were observed, and that had not had human assistance to deliver their lambs. The effects of body condition and nutrition on the behaviour of these ewes and lambs were minor and have been reported elsewhere (Table 1). Combining data from all seven studies provided statistical power to analyse the association between individual lamb and ewe behaviours post-lambing and lamb survival to weaning. To date the majority of studies of the association of ewe and lamb behaviour with lamb survival, particularly under extensive pastoral conditions, have contained too few animals to allow for conclusions to be drawn.

### Behavioural measurements in the paddock

In each study listed in Table 1, ewes were inspected twice daily during the lambing period at 8 am and 4 pm to identify newly born lambs. Lambs were handled if their coat was dry and all lambs in the litter were mobile (lambs were between three and 18 hours of age, although their exact age was not known). During handling, lambs were identified to their dam, ear-tagged and had their live weight, birth-rank and sex recorded. During the handling of the lambs the maternal behaviour score (MBS) of the ewe was assessed. MBS was scored on a five-point scale based on the distance the ewe moved away from her lambs while they were being handled (one, ewe flees and does not return; five, ewe remains within one metre of the lambs and makes contact with either the lamb or shepherd; [11]).

At the completion of handling the final lamb of a litter, all lambs were placed together on the ground, lying on their side, while three observers moved approximately 10 metres away. Observers recorded the individual behaviours of each of the lambs and ewe for next five minutes. Release of the lambs was considered to be ‘time zero’. The lamb behaviours recorded included: the time at which the lamb stood (defined as fully supporting itself on all four legs for at least five seconds), the ewe and lamb made contact (defined as being within 0.5 metres of each other [9,14], the lamb followed the ewe for at least five metres from their first point of contact [9] and the lamb successfully sucked from its dam’s teat (lamb held teat in its mouth and appeared to be sucking with for at least five seconds). A binary value was given for whether a behaviour trait was expressed during the observation period. Lambs that expressed a behaviour were given a value of 1 and those that did not show the behaviour a value of 0. In addition, the number of seconds from the start of observation until the behaviour was observed was recorded. Observers also counted the number of low-pitched bleats (bleats involving little mouth movement) and high-pitched bleats (bleats involving full mouth movement [9] emitted by each lamb and ewe during the 5-minute observation period.

### Maternal-recognition test

**Figure 1:**
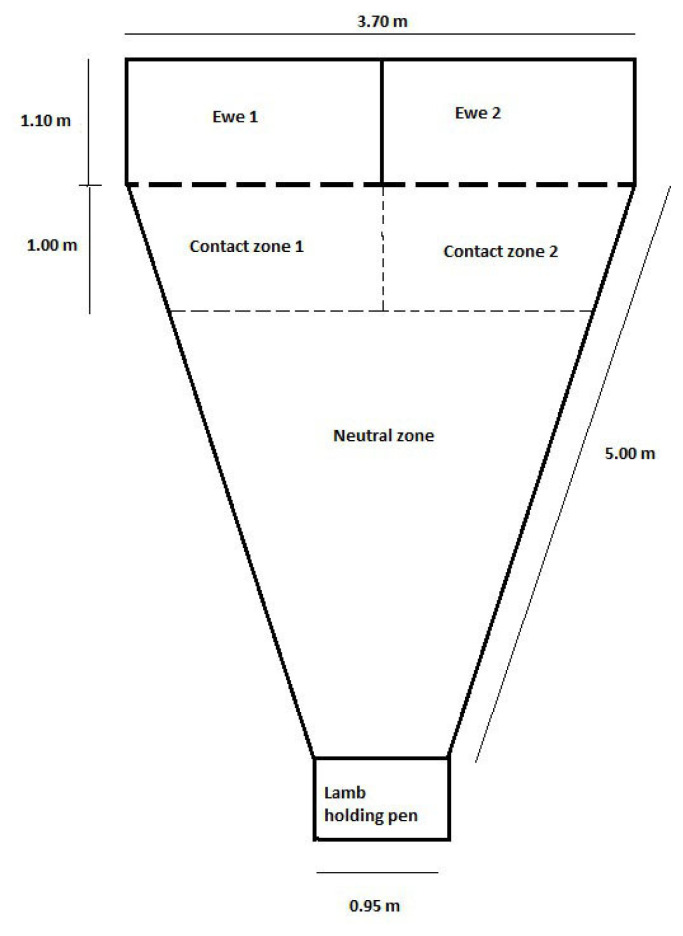
Layout of testing arena and holding pens for the maternal-recognition test. Adapted from [28].

A maternal-recognition test (MRT), as described by Nowak et al [15,16] was conducted in three of the seven studies (Table 1). The test was conducted at approximately 12 or 24 hours after birth on a subset of litters that had been observed in the paddock (twins lambs at 12 h n = 130 and at 24 h n = 60; triplet lambs at 12 h n = 273; and at 24 h n = 134) in each study. Testing occurred between 1 pm and 3 pm daily. The subset was a convenience sample (based on lambs being either 12 or 24 hours of age at the time that the testing arena was available). The ability of the lamb to discriminate its dam from an alien ewe has previously been used as an indicator of the strength of the ewe-lamb bond (Nowak et al [15,16], but cannot be easily evaluated during paddock observations. The testing arena was of a triangular shape fenced with solid one metre high walls (3.7×6.1 m, Figure 1). Adjacent to the vertex of the triangle pen was a lamb holding pen. At the base of the triangle arena there were two ewe pens (1.85×1.1 m), separated from the testing arena by wire-mesh gates. The arena was divided into three zones separated by lines painted on the ground; a neutral zone (the area of the triangle that was at least one metre from the ewe pens), and two ewe contact zones adjacent to each of the two ewe pens (Figure 1).

The dam of the lamb being tested was placed randomly in one of the two ewe pens, with an ‘alien’ ewe which had lambed at a similar time placed in the other pen. Each lamb was tested individually and was placed, standing, in lamb holding pen facing the two ewe pens. Once the lamb was released into the neutral zone the lamb could see both ewes in the pens. All other lambs, including the sibling of the lamb being tested and the lambs of the alien ewe were kept approximately five metres away so that the ewes and test lamb could hear but not see them. Each lamb was only tested once to avoid any possible effects of learning [16].

Each MRT was conducted for five minutes and the location and activity of the lamb was recorded at 10 second intervals. This allowed for an estimation of the total time that the lamb spent: in each zone of the arena, and the time spent sitting, standing and walking to be calculated. The lamb was considered to be preferentially attracted to one of the ewes if it spent at least two-thirds of the time in the contact area adjacent to the ewe [16]. There were three possible outcomes of this preference test; correct choice; the lamb entered the contact zone and spent at least two-thirds of this time beside its dam; incorrect choice; the lamb entered the contact zone and spent two-thirds of this time beside the alien ewe; and *no choice*; the lamb spent more than one-third but less than two-thirds of its time with each ewe [16]. High and low-pitched bleats emitted by the lamb were counted for the duration of the test. A binary value was also recorded for whether or not a behaviour traits including walking, standing, sitting, reached dam, reached contact zone, spent time with dam, spent time with alien and spent time in contact zone was observed during the five minute period.

Lambs were weaned at a mean age of between 70 and 111 days of age (Table 1). Survival of the lamb to weaning was determined based on its presence or absence of the lamb at weaning.

### Statistical analysis

Statistical analyses were conducted using SAS v9.3 (SAS Institute Inc., 2011; Cary, NC, USA). Twin- and triplet-born lambs were analysed separately. Descriptive statistics including the median were generated for each behaviour trait.

The base model was a logistic regression based on a logit transformation with survival to weaning as the binomial outcome variable. The model included the experiment identifier and sex of lamb as fixed effects. Birth weight of lamb was included as a covariate. The quadratic effect of birth weight was tested but was found to be non-significant (p>0.05) and therefore, was removed from the final models. The models for MRT traits also included the approximate age of the lamb (12 h or 24 h) as a fixed effect.

Each lamb or ewe behaviour trait was fitted in the logistic regression model independently. The binary expression of a trait was fitted as a fixed effect in a model that included all lambs of the relevant birth-rank. The continuous variables of time to exhibit a trait, the duration that the behaviour was exhibited, or number of bleats, were fitted as covariates in models that included all the lambs of the relevant birth-rank that displayed the trait (i.e. binary value of 1). Outcome of the MRT (i.e. correct choice, incorrect choice or no choice) and MBS were tested as fixed effects. The odds ratios are presented with 95% confidence intervals (CI) given in parentheses.

## RESULTS

### General

**Figure 2:**
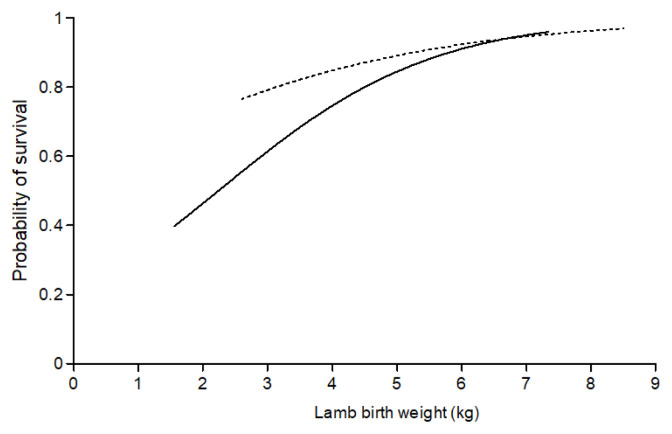
The effect of lamb birth weight (kg) on the probability of survival for twin-(- -) and triplet-born (—) lambs (values are back-transformed).

Lamb survival from tagging to weaning across all studies ranged from 82.5% to 90.3% for twin- and 73.0% to 76.0% for triplet-born lambs (Table 1). Birth weight and sex of the lamb influenced survival of both twin- and triplet-born lambs. For every additional 100 g of birth weight twin- and triplet-born lambs were 1.468 (95% CI, 1.102 to 1.956, p<0.05) and 1.840 (1.497 to 2.286, p<0.05) times more likely to survive, respectively (Figure 2). Female lambs were more likely (p< 0.05) to survive than male lambs (odds ratio 1.552 [1.002 to 2.404] for twin-born lambs and 1.599 [1.152 to 2.225] for triplet-born lambs).

### Maternal behaviour score and ewe and lamb vocalisation in the paddock

Maternal behaviour score was not associated (p>0.05) with the survival of twin- or triplet-born lambs (odds ratio 1.022 [95% CI 0.844 to 1.237] and 1.029 [95% CI 0.888 to 1.192], respectively).

The odds of surviving to weaning were similar (p>0.05) for twin- and triplet-lambs that bleated in a high or low pitch compared with those that did not bleat during the observation period (data not shown). Similarly, whether their dam bleated or not during the observation period had no effect on twin- or triplet-lamb survival (data not shown). Survival to weaning of twin-born lambs was not affected by the number of high- or low-pitched bleats emitted, however, triplet-born lambs were 1.059 (95% CI 1.007 to 1.112) times more likely to survive for every extra low-pitched bleat they emitted (Table 2). The number of high- and or low-pitched bleats emitted by the ewe showed no relationship with lamb survival for either twin- and triplet-born lambs (p>0.05, Table 2).

### Lamb behaviours in the paddock and lamb survival

There was no relationship between lamb survival to weaning and whether or not twin- and triplet-born lambs stood, made contact with their dam, sucked from their dam or followed their dam (data not shown). Similarly, the time taken for lambs to stand, make contact with and suck from their dam was not associated with lamb survival for either twins or triplets (Table 3). Twin-born lambs were 1.004 (95% CI 1.000 to 1.008) times more likely to survive for every second longer it took them to follow their dam, whereas triplet-born lambs were 0.996 (95% CI 0.993 to 0.999) times as likely to survive for every second longer.

### Lamb behaviour during the maternal-recognition test

The odds of a lamb surviving to weaning were not influenced by whether the twin- or triplet-born lambs were observed to walk, stand, sit, reach the contact zone, reach their dam, spend time with their dam or an alien ewe, or bleat in a high- or low-pitch during the maternal recognition test (p>0.05, Table 4). There were also no association between the amount of time that the lamb spent walking, standing, sitting, in the contact zone, with an alien ewe or with their dam and the odds of survival to weaning (p>0.05, Table 5).

For every additional second twin-born lambs took to reach the contact zone, they were 0.989 (0.979 to 1.000) times less likely to survive to weaning (p<0.05), however, the time required for twin-born lambs to reach their dam was not associated with the odds of survival (p>0.05; Table 5). Twin-born lambs were 0.987 (0.964 to 0.992) times less likely to survive to weaning (p<0.05) for each additional high-pitched bleat they expressed, however, there was no relationship between survival to weaning and number of low-pitched bleats (p>0.05).

Among triplet-born lambs, the odds of survival were not influenced (p>0.05) by the amount of time the lamb spent walking, standing, sitting, in the contact zone, with the alien ewe or with their dam (Table 4). For every second longer that a triplet-born lamb took to reach its dam it was 0.994 (0.989 to 0.999) times less likely to survive (p<0.05). The number of high- and low-pitched bleats was not associated with the odds of a triplet-lamb surviving to weaning (p>0.05).

The outcome of the MRT (correct 55% and 48%, incorrect 19% and 22% or no choice 26% and 30% for twin- and triplet-born lambs, respectively) was not associated with lamb survival to weaning for twin- or triplet-born lambs (data not shown, p>0.05).

## DISCUSSION

Mortality rates in this study were within the range of those previously reported for both twin- (8.1% to 26.7%) and triplet-born (12.7% to 53.4%) lambs managed under extensive pastoral systems in New Zealand [2,17]. The relationship between the sex of the lamb [18] and a curvilinear relationship of lamb birth weight [19,20] with survival to weaning were also consistent with previous work. Interestingly, data from the present study suggests that at low birthweights triplet lambs have lower survival than twins but this is not seen at heavier birth weights. This suggests that the mechanism that increases birth weight also has a positive effect on triplet lamb survival.

### Paddock behaviours

Vocalisation of the ewes in the paddock was not associated with the odds of twin- and triplet lambs surviving to weaning. In addition, ewe MBS measured at approximately 3 to 18 hours after birth was not associated with lamb survival. This finding is contrary to previous studies of Lambe et al [21] who reported that more lambs born to ewes with a MBS of one, compared with those born to ewes with higher scores, died before weaning. Similarly, Everett-Hincks et al [22] reported that litter survival increased for dams that had a MBS of three compared with dams with an MBS of one or two. Plush et al [10] also reported a positive correlation between ewe MBS and lamb survival. The reason for the differences in findings of these studies and the current study is unknown. In the current study, Romney cross ewes were utilised compared with the previous studies that used Scottish Blackface, Merino and Coopworth ewes. Maternal behaviour is known to vary among breeds for example time spent at the lambing site and grooming behaviour [3]. In addition, the previous studies included a mix of single-, twin- and triplet-born lambs while in the current study only twin- and triplet-lambs were enrolled. The results from the current study, however, indicate that the behaviour of the ewe 3 to 18 hours after birth was not a useful predictor of lamb survival to weaning. Similarly, Dwyer et al [23] hypothesised that variation in the quantity or quality of maternal behaviour expressed may not have a great influence on lamb survival as lamb survival had a negligible correlation with maternal attachment scores and poor genetic correlation with maternal behaviour.

The majority of twin- and triplet-lamb behaviours observed at ear tagging were not related to survival to weaning. This suggests that behaviours such as standing, ewe-lamb contact, sucking, following and bleating at approximately 3 to 18 hours after birth are not suitable as potential indicators of the likelihood of survival to weaning. Triplet-born lambs that emitted more low-pitch bleats, however, had better odds of survival than those bleated less. Low-pitched bleats are emitted almost exclusively between the neonates and their dam and help maintain the bond between the ewe and her lambs [24]. The increased frequency of low-pitched bleats and improved survival, may be reflective of a more secure ewe-lamb bond, or alternatively, may stimulate the development of a more secure ewe-lamb bond that leads to increased survival to weaning. It has been suggested that triplet-rearing ewes are poorer at communicating with their lambs when separated from some of her lambs compared with twin-rearing ewes [25]. It is possible that a triplet-lamb that bleats more may be more likely to attract the attention of its dam and thus be reunited increasing its chance of survival.

Twin-bearing ewes produce roughly 40% more milk than single-bearing ewes, but triplet-bearing ewes produce only up to 10% more milk than twin-bearing ewes [26]. Hinch et al [27], calculated that twin lambs have a mean milk intake of 59.9% of dingles and triplets 69.8% that of twins. As such, there is a significantly greater competition for milk within triplet litters compared with twin litters. This may explain why triplet-born lambs had an increased odds of survival if they are quicker to follow their dam. Interestingly, in twin-born lambs, lambs that followed their dam more quickly had a lower the probability of survival to weaning than lambs that were slower to follow. Hinch et al [27], however, estimated that at one week of age single, twin- and triplet-rearing ewes produced more milk than was demanded by the lamb(s) thus milk production is unlikely to have a significant influence on lamb behaviour at 3 to 18 hours of age. The reason for this difference is unclear and requires further investigation. Time to follow is a measure of how long the lambs took to follow their dam at least 5 m from the point of contact. This means a longer time to follow was recorded when either the ewe moved away and the lambs took some time to follow her, or when the ewe remained at the contact site tending to her lambs for some time before moving away with her lambs. Therefore, the negative relationship between time to follow and survival in twin-born lambs may be explained by more attentive mothers contributing to an extended time to follow.

In the present study, lamb behaviours were observed at tagging, when lambs were approximately 3 to 18 hours of age, so it may not be surprising that the results observed differ from previous research that examined ewe and lamb behaviours immediately after birth [3,6]. In the present study, a minimum of three hours had elapsed after birth before recording behaviour occurred to ensure that ewe and lamb bonding had occurred. As a consequence, any lambs that had died prior to tagging, and their siblings, were excluded from the study. This may have unintentionally excluded very weak lambs and those born to dams of low MBS from the data collection. It is likely that the majority of lambs that died within the first three hours after birth would have been a result of dystocia as there was insufficient time for starvation/exposure to be the cause of death [17].

### Maternal-recognition test

Nowak and Lindsay [28] reported that single- and twin-born Merino lambs that survived past seven days of life spent more time with their dam when tested at 12 hours of age, but found no association when lambs were tested at 72 hours of age. In the present study, however, there was no association between time spent with the dam and survival for twin- or triplet-born lambs at either 12 or 24 hours. It is possible that differences could be due to breed differences. In addition, Nowak and Lindsay [28] measured lamb survival until only seven days of age, while the present study measured survival to weaning. Regardless of the causes of difference the current results indicate that time spent near the dam in the MRT as conducted in the current study was not a suitable measure of lamb survival.

Triplet-born lambs that reached their dam more quickly during the maternal recognition test had a better odds of survival to weaning than those that reached their dam more slowly. Among twin-born lambs, simply reaching the contact zone increased their odds of survival. These measures may be proxies for vigour or indicators of the strength of the bond between the ewe and her lambs. Pollard [25] observed that the response of ewes to one absent or separated lamb decreased as the number of lambs being reared increased hypothesising that this was a result of the ewe having more stimuli from the remaining lambs. Triplet lambs, therefore, may have to play a greater role in maintaining contact with their dam than twin lambs. Although significant, the size of the effect was small, so that it is unlikely to be a reliable screening tool to determine whether intervention is required. Furthermore, as it requires the use of a testing arena and thus would likely only be applicable in a research situation.

In the MRT twin-born lambs that had a greater frequency of high-pitched bleats had a lower survival rate than those that bleated less frequently. High-pitched bleats are considered a distress bleat and are frequently expressed when the lamb and ewe are separated [12,29]. Lambs expressing these high-pitched bleats may have had a less secure bond with their dam. It would be expected, however, the relationship between the frequency of high-bleats and lamb survival would be similar between twin and triplet lambs. Corner et al [30] reported that a similar proportion of twin and triplet lambs were observed to bleat and at a similar frequency, although in that study low and high pitch bleats were not recorded separately. It is possible that the larger proportion of triplet lambs that died compared with twins may have altered the relationship between high bleat frequency and survival. During the maternal recognition test, vocalisations of the ewe and lamb were not recorded, therefore, it was not possible to determine whether lamb high-pitched bleats were emitted in response to the dam or independently. In addition, during the paddock observations there was no relationship between the frequency of high bleats emitted by the lamb and their survival to weaning. This suggests that this may be a spurious finding and further research is necessary to examine this relationship.

## CONCLUSION

The majority of the behaviours measured in the paddock were not associated with lamb survival to weaning, and those that were had only a minor effect. These behaviours therefore cannot be used by farmers with confidence to determine the lambs that require intervention to improve their chances of survival. During the MRT, time to reach dam or time to reach contact zone for triplet-born or twin-born lambs, respectively, showed an association with survival, but again, the effect was not large enough to provide a useful prediction of survival. These results suggest that observations made outdoors and with lambs ranging from 3 to 18 hours age may not provide a sufficiently controlled environment to allow the identification of lambs at risk of mortality. Alternative measures of behaviour, or behaviour relative to other lambs in the litter, therefore, are required in order to identify predictors for survival.

## Figures and Tables

**Table 1:** Summary of studies from which behaviour data was collected1)

Paddock behaviour reported	Year of study	Birth rank	Treatments2)	n3)		MRT	Lamb age at weaning	Lamb survival (%)

				Ewes	Lambs			
Gronqvist [31]	2010	Twin	M/A P112 to P136	154	308	Yes	82	82.5
Gronqvist [31]	2011	Twin	M/A P128 to P141	120	240	No	87	92.9
Gronqvist [32]	2011	Twin	R/M/H P141 to L79	92	184	No	79	90.2
Gronqvist [33]	2013	Twin	M/A P76 to L1	57	114	No	111	90.3
Gronqvist [34]	2009	Triplet	M/A P93 to P114	88	264	Yes	70	73.1
Gronqvist [35]	2010	Triplet	M/A P115 to P136	119	357	No	91	75.1
Gronqvist [35]	2011	Triplet	M/A P128 to P142	88	264	Yes	80	76.0

BCS, body condition score; MRT, maternal recognition test.

1) Source of data for analyses examining of the impact of the ewe and lamb behaviour on lamb survival to weaning showing year of study, the birth rank investigated, nutritional treatments, the citation for the report of the impact of ewe nutrition and BCS on ewe and lamb behaviour, number of ewes and lambs with behavioural observations, whether a MRT was conducted, the age of the lamb at weaning and the overall survival rate of lambs with behavioural observations.

2) R, restricted (800 to 1,000 kg DM/ha); M, medium (1,000 to 1,200 kg DM/ha); A, *ad libitum* (>1,200 kg DM/ha); H, high (1,500 to 1,700 kg DM/ha); P, mean day of pregnancy; L, mean day of lactation.

3) Number of ewes and lamb with behaviour observations.

**Table 2:** Twin- and triplet-lamb bleating behaviour1)

Bleat type	Birth rank	n2)	%	Median bleats (n)	Odds ratio	p-value
Lamb						
High-pitch	Twin3)	726	85.8	10	0.996 (0.983–1.009)	0.557
Low-pitch	Twin3)	694	82.0	5	0.992 (0.969–1.015)	0.485
High-pitch	Triplet4)	708	80.0	6	0.994 (0.986–1.003)	0.169
Low-pitch	Triplet4)	495	55.9	15	1.059 (1.007–1.112)	0.024
Ewe						
High-pitch	Twin3)	726	85.8	13	0.998 (0.980–1.017)	0.865
Low-pitch	Twin3)	752	88.9	3	0.999 (0.988–1.010)	0.862
High-pitch	Triplet4)	699	79.0	8	1.006 (0.993–1.020)	0.354
Low-pitch	Triplet4)	731	82.5	20	1.002 (0.994–1.010)	0.597

CI, confidence intervals.

1) The number and percentage of lambs that bleated or whose dam bleated during the observation period, the median number of bleats recorded and the effect of the number of bleats on the odds (95% CI) for survival to weaning.

2) The number of lambs that were recorded to bleat, or the number of lambs whose dam bleated at least once.

3) There were a total of 846 twin lambs observed across four studies.

4) There were a total of 885 triplet lambs observed across three studies.

**Table 3:** Twin and triplet lamb behaviours observed 3 to 18 hours after birth1)

Items	n2)	%	Median time	Odds ratio3)	p-value
Twin-born lambs					
Stand	780	92.2	20	1.001 (0.997–1.005)	0.603
Contact	776	91.7	14	1.002 (0.997–1.006)	0.482
Suck	239	28.3	139	1.003 (0.997–1.010)	0.282
Follow	512	60.5	120	1.004 (1.000–1.008)	0.031
Triplet-born lambs					
Stand	731	82.6	25	0.999 (0.996–1.002)	0.507
Contact	711	80.3	16	1.000 (0.997–1.003)	0.962
Suck	159	18.0	165	0.998 (0.995–1.007)	0.674
Follow	361	40.8	120	0.996 (0.993–0.999)	0.007

CI, confidence intervals.

1) The number of twin- and triplet-lambs that expressed each behaviour in the paddock during the five-minute observation period (n), the median time (seconds) taken to express the behaviour and the effect of the time taken on the odds (95% CI) of survival to weaning.

2) The total number of lambs that exhibited the behaviour.

3) Odds ratio (95% CI).

**Table 4:** Behaviours expressed by twin and triplet lamb during the maternal recognition test1)

Behaviour	n	%	Odds ratio2)	p-value
Twin-born lambs3)				
Walked	182	95.8	1.002 (0.117–8.590)	0.998
Stood	188	98.9	6.177 (0.360–105.971)	0.209
Sat	66	34.7	2.263 (0.801–6.391)	0.123
Reached dam	160	83.7	1.377 (0.469–4.045)	0.560
Reached contact zone	178	93.7	2.280 (0.567–9.173)	0.245
Spent time with dam	160	83.7	1.455 (0.493–4.293)	0.497
Spent time with alien	144	75.8	1.053 (0.388–2.856)	0.919
Spent time in contact zone	179	94.2	2.609 (0.637–10.696)	0.182
High-pitch	186	97.9	3.636 (0.308–42.930)	0.305
Low-pitch	133	70.0	0.527 (0.182–1.522)	0.236
Triplet-born lambs4)				
Walked	389	94.9	1.661 (0.552–4.992)	0.366
Stood	404	98.5	1.645 (0.144–18.784)	0.688
Sat	65	15.9	0.602 (0.293–1.235)	0.166
Reached dam	363	88.5	0.8325 (0.241–2.047)	0.462
Reached contact zone	391	95.4	1.044 (0.276–3.955)	0.948
Spent time with dam	363	88.5	1.352 (0.785–2.328)	0.693
Spent time with alien	346	84.4	0.841 (0.376–1.883)	0.673
Spent time in contact zone	391	95.4	1.044 (0.276–3.955)	0.948
High-pitch	398	97.1	6.003 (0.960–37.531)	0.055
Low-pitch	250	61.0	1.158 (0.668–2.009)	0.601

CI, confidence intervals.

1) The percentage of twin- (n = 190) or triplet-born (n = 410) lambs that expressed each behaviour during the maternal-recognition test and the effect of exhibiting the behaviour on the odds (95% CI) of survival to weaning.

2) The effect of behaviour on the probability of survival to weaning is presented as the point estimate (with the 95% confidence limit in parenthesis) followed by the p-value.

3) There was a total of 190 twin lambs observed during one study.

4) There was a total of 389 triplet lambs observed across two studies.

**Table 5:** Duration of behaviours of twin and triplet lamb during the maternal recognition test1)

Behaviour	n2)	Median time3)	Odds ratio4)	p-value
Twin-born lambs				
Walked	182	50	1.001 (0.990–1.011)	0.905
Stood	188	240	0.996 (0.987–1.005)	0.358
Sat	66	30	0.993 (0.983–1.004)	0.224
Reached dam	160	30	0.999 (0.991–1.008)	0.838
Reached contact zone	178	20	0.989 (0.979–1.000)	0.047
Spent time with dam	160	170	1.002 (0.997–1.006)	0.431
Spent time with alien	144	70	1.001 (0.996–1.006)	0.718
Spent time in contact zone	179	280	1.003 (0.999–1.008)	0.157
Triplet-born lambs				
Walked	389	50	1.001 (0.995–1.007)	0.748
Stood	404	240	1.002 (0.997–1.007)	0.348
Sat	65	60	0.996 (0.990–1.002)	0.173
Reached dam	363	30	0.994 (0.989–0.999)	0.029
Reached contact zone	391	20	0.991 (0.981–1.002)	0.098
Spent time with dam	363	170	1.001 (0.998–1.004)	0.449
Spent time with alien	346	80	1.001 (0.997–1.004)	0.742
Spent time in contact zone	391	280	1.003 (0.999–1.006)	0.151

CI, confidence intervals.

1) The number of twin- (n = 190) and triplet-lambs (n = 410) that expressed each behaviour during maternal-recognition test, the median time (seconds) taken to express the behaviour and the effect of the median time taken on the odds (95% CI) of survival to weaning.

2) The number of lambs that express the behaviour trait.

3) The median time in seconds taken to express a behaviour or the median time spent expressing and behaviour during the maternal-recognition test.

4) The effect of lamb behaviour on the probability of survival to weaning is presented as the point estimate (with the 95% confidence limit in parenthesis) followed by the p-value.
